# Bacterial pneumonia patients with elevated globulin levels did not get infected with SARS-CoV-2: two case reports

**DOI:** 10.3389/fimmu.2024.1404542

**Published:** 2024-08-29

**Authors:** Qi Zhong, Qiu-mei Lin, Hong-bin Long, Cai-xia Liao, Xiao-xiao Sun, Miao-du Yang, Zhi-hao Zhang, Yi-hua Huang, Shi-min Wang, Zhao-shou Yang

**Affiliations:** ^1^ The First Affiliated Hospital/The First School of Clinical Medicine of Guangdong Pharmaceutical University, Guangdong Pharmaceutical University, Guangzhou, Guangdong, China; ^2^ Zhongshan School of Medicine, Sun Yat-Sen University, Guangzhou, China

**Keywords:** bacterial pneumonia, globulin protein, SARS-CoV-2, cross-reactivity, case reports

## Abstract

**Background:**

COVID-19 began in December 2019, rapidly spreading worldwide. China implemented a dynamic zero-COVID strategy and strict control measures after the outbreak. However, Guangzhou city ended closed-off management by the end of November 2022, leading to exposure to SARS-CoV-2. Despite most hospitalized patients being infected or co-infected with SARS-CoV-2, some remained uninfected. We report two cases of bacterial pneumonia with elevated globulin levels not infected with SARS-CoV-2, aiming to identify protection factors of SARS-CoV-2 infection and provide a scientific basis for SARS-CoV-2 prevention.

**Case presentation:**

Case 1, a 92-year-old male, admitted on October 21, 2022, developed worsening cough and sputum after aspiration, diagnosed with bacterial pneumonia with Pseudomonas aeruginosa, Escherichia coli (CRE) and carbapenem-resistant Acinetobacter baumannii (CRAB) infections. He was treated with imipenem anti-infective therapy and mechanical ventilation, then switched to a combination of meropenem, voriconazole and amikacin anti-infective therapy due to recurrent infections and septic shock, and died of sepsis on 8 January 2023. Case 2 is an 82-year-old male admitted on 30 September 2022, with recurrent cough, sputum, and shortness of breath, diagnosed with bacterial pneumonia with carbapenem-resistant Klebsiella pneumoniae (CRKP) and Mycobacterium pneumoniae infections. He was treated with ventilator-assisted ventilation, meropenem, amikacin, tigecycline and mucomycin nebulization and discharged with improvement on 26 October. He was readmitted on 21 November 2022 and diagnosed with bacterial pneumonia. He was treated with cefoperazone sulbactam, amikacin, meropenem and fluconazole and discharged on 31 December. Neither patient was infected with SARS-CoV-2 during hospitalization. Notably, their globulin levels were elevated before SARS-CoV-2 exposure, gradually decreasing afterward.

**Conclusions:**

Patients with bacterial pneumonia with high globulin levels likely have large amounts of immunoglobulin, and that immunoglobulin cross-reactivity causes this protein to be involved in clearing SARS-CoV-2 and preventing infection. Therefore, bacterial pneumonia patients with high globulin levels included in this study were not infected with SARS-CoV-2. After exposure to SARS-CoV-2, the amount of globulin in the patient’s body was reduced because it was used to clear SARS-CoV-2. The results of this study are expected to provide a theoretical basis for the study of the mechanism of prevention and treatment of SARS-CoV-2 infection.

## 1 Introduction

COVID-19, caused by SARS-CoV-2, first emerged in Wuhan, China, in late 2019 and then rapidly spread worldwide to become a pandemic (1, 2). China adopted a dynamic zero-COVID strategy to prevent and control the outbreak by the end of November 2022 (3). Subsequently, China optimized its control measures, including implementing ‘20 measures’ (4) and lifting closed-off management measures (5). Guangzhou ended closed-off management on 30 November 2022 (6). This led to hospitalized patients beginning to be exposed to an environment filled with SARS-CoV-2, ultimately leading to an outbreak of SARS-CoV-2 infections in December 2022 in Guangzhou (7, 8). However, the results of our previous study found that some of the patients with bacterial pneumonia in December 2022 were not infected with SARS-CoV-2 during their hospitalization (7).

Bacterial pneumonia is a lung disease caused by bacterial infection, and common pathogens include Streptococcus pneumoniae, Staphylococcus aureus, Pseudomonas aeruginosa, Klebsiella pneumoniae, and Haemophilus influenzae (9, 10). Typical clinical symptoms include fever, chills, cough (may cough up purulent sputum), chest pain, and shortness of breath and dyspnoea. Diagnosis can be made by physical examination, medical history, chest X-ray (CXR) and laboratory tests (sputum culture and blood tests). Treatment is initially based on empirical antibiotics, with subsequent adjustments to the regimen based on sputum culture and drug susceptibility testing, supplemented by supportive therapy such as adequate hydration, oxygenation, and physical expectoration. While the prognosis for most patients is good, if the prognosis is poor, sepsis and infectious shock may develop. Currently, though many studies focused on co-infection with bacteria in patients with COVID-19 (11, 12), there are relatively few studies and reports on the susceptibility of patients with bacterial pneumonia to SARS-CoV-2.

This study describes two hospitalized patients with bacterial pneumonia who were not infected with SARS-CoV-2. Their globulin levels were above the upper limit of the reference value, but gradually decreased and remained above the lower limit of the reference value after lifting closed-off management. This study is expected to identify protection factors of SARS-CoV-2 infection and provide a scientific basis for its prevention and treatment.

## 2 Case presentation

### 2.1 Case 1 presentation

On 21 October 2022, a 92-year-old male was admitted to our Rehabilitation Unit with progressive memory loss, diagnosed with Alzheimer’s disease and treated with olanzapine, electroencephalogram (EGG) biofeedback, and cognitive training. He was transferred to ICU on 17 November 2022, with worsening cough and sputum and shortness of breath due to aspiration of bloody fluid from the nasal passage. He had a history of left-sided nosebleed, malignant melanoma with multiple metastases in the left nasal cavity for over 2 years, hypertension, diabetes mellitus, and history of transfusion of AB Rh+ red blood cell suspension without transfusion reaction. He denied history of infectious diseases such as hepatitis and tuberculosis, drug and food allergies. He denied any close contact with history of SARS-CoV-2 infection.

**Figure 1:**
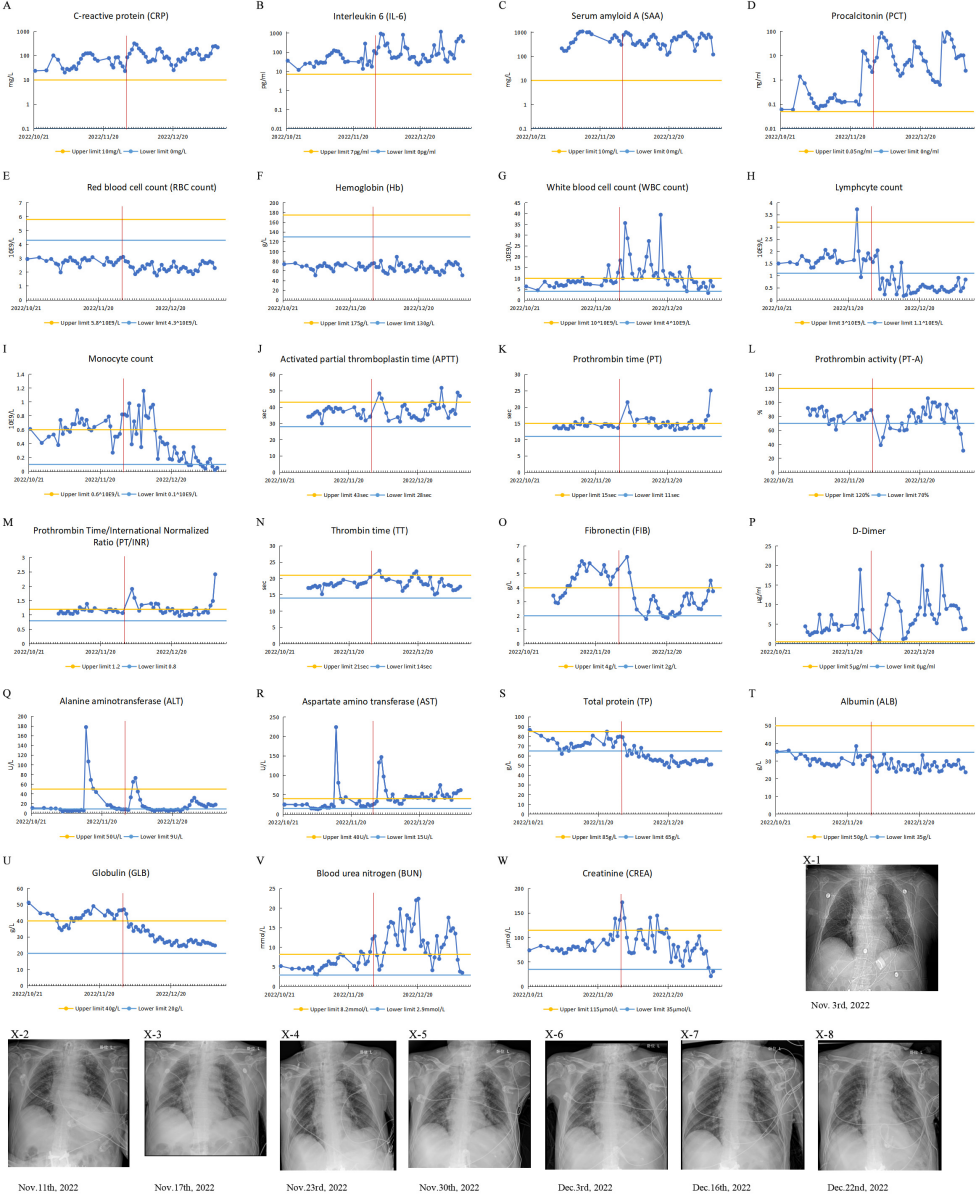
Clinical test results of case 1 from October 21, 2022, to January 9, 2023. The patient’s IL-6 and PCT were measured with a Roche cobas e 601. CRP, SAA, TP, Alb, Glob, AST and ALT were measured with a BECKMAN COULTER AU5800. The patient’s Routine blood tests were measured with a Mindray BC-6900 instrument. **(A-W)** Test results of patient’s blood specimens. The horizontal axis represents the dates, and the solid blue dots indicate the test results at the corresponding time points. The red vertical line in the figure corresponds to November 30, 2022, when the city of Guangzhou ended closed-off management. **(A-D)** Infection-related test results: CRP, IL-6, PCT and SAA. **(E-I)** Routine blood test results: RBC, Hb, WBC, monocyte count, lymphocyte count. **(J-P)** Coagulation-related test results: APTT, PT, PT-A, PT/INR, TT, FIB and D-Dimer. **(Q, R)** Liver function-related test results: ALT and AST. **(S-U)** Serum protein-related test results: TP, ALB and GLB. **(V, W)** Renal function-related test results: BUN and CREA. **(X1-8)** CXR test results.

He was diagnosed with bacterial pneumonia with the presence of Pseudomonas aeruginosa, Escherichia coli (CRE), and carbapenem-resistant Acinetobacter baumannii (CRAB) infections. His physical examination revealed coarse breath sounds in both lungs, accompanied by a significant amount of dry and moist rales. Some of the clinical laboratory findings are shown in **Figure 1**. The examination results suggested leukocytosis, neutrophilia, elevated infection markers, sputum smear suggestive of gram-negative bacilli, and sputum culture suggestive of Pseudomonas aeruginosa, *Escherichia coli* (CRE), and CRAB. The patient’s CXR showed thickening and blurring of the texture of both lungs, and scattered few patches of patchy, reticulated, or flocculent slightly dense blurred shadows were seen in both lungs. Sputum smear and CXR supported the diagnosis of pneumonia. The ORF1ab Gene and N Gene assays for the 2019-nCoV-RNA were performed using RT-qPCR, and he was tested negative for the SARS-CoV-2 during hospitalization.

He was treated with piperacillin sodium sulbactam sodium for anti-infection, ambroxol for sputum, and intensive bedside suctioning. Later, considering the recurrent infection, he was switched to imipenem anti-infective treatment, mechanical ventilation, and continuous renal replacement therapy (CRRT) if necessary. On 19 December, due to the patient’s recurrent infection and septic shock, the anti-infective treatment regimen was adjusted, and he was given a combination of meropenem, voriconazole, and amikacin for anti-infective treatment. Unfortunately, the patient died of sepsis on 8 January 2023.

It is worth noting that the patient was not infected with SARS-CoV-2 during hospitalization. When he was admitted to the hospital, his peripheral blood globulin level was significantly elevated, exceeding the upper limit of the reference range. During hospitalization, although his globulin levels occasionally fell below the upper limit, they remained high. On November 30, 2022, the day of the lifting of closed-off management, his peripheral blood globulin level was still above the upper limit of the reference range. Following that, the level gradually decreased but remained above the lower limit of the reference range.

### 2.2 Case 2 presentation

On 30 September 2022, an 82-year-old male was admitted to our respiratory department with recurrent cough, sputum, and shortness of breath. Over a year ago, he coughed up sputum for no apparent cause and was diagnosed with a lung infection, which was ultimately diagnosed as severe pneumonia and treated at several hospitals. He had a history of cerebral infarction for more than 6 years, with inability to cough up sputum autonomously; history of various chronic diseases and hypertension. He denied the history of tuberculosis, surgery, trauma, blood transfusion, and allergy. He denied any close contact with history of SARS-CoV-2 infection. His personal and family history, medication history and social history were normal.

**Figure 2:**
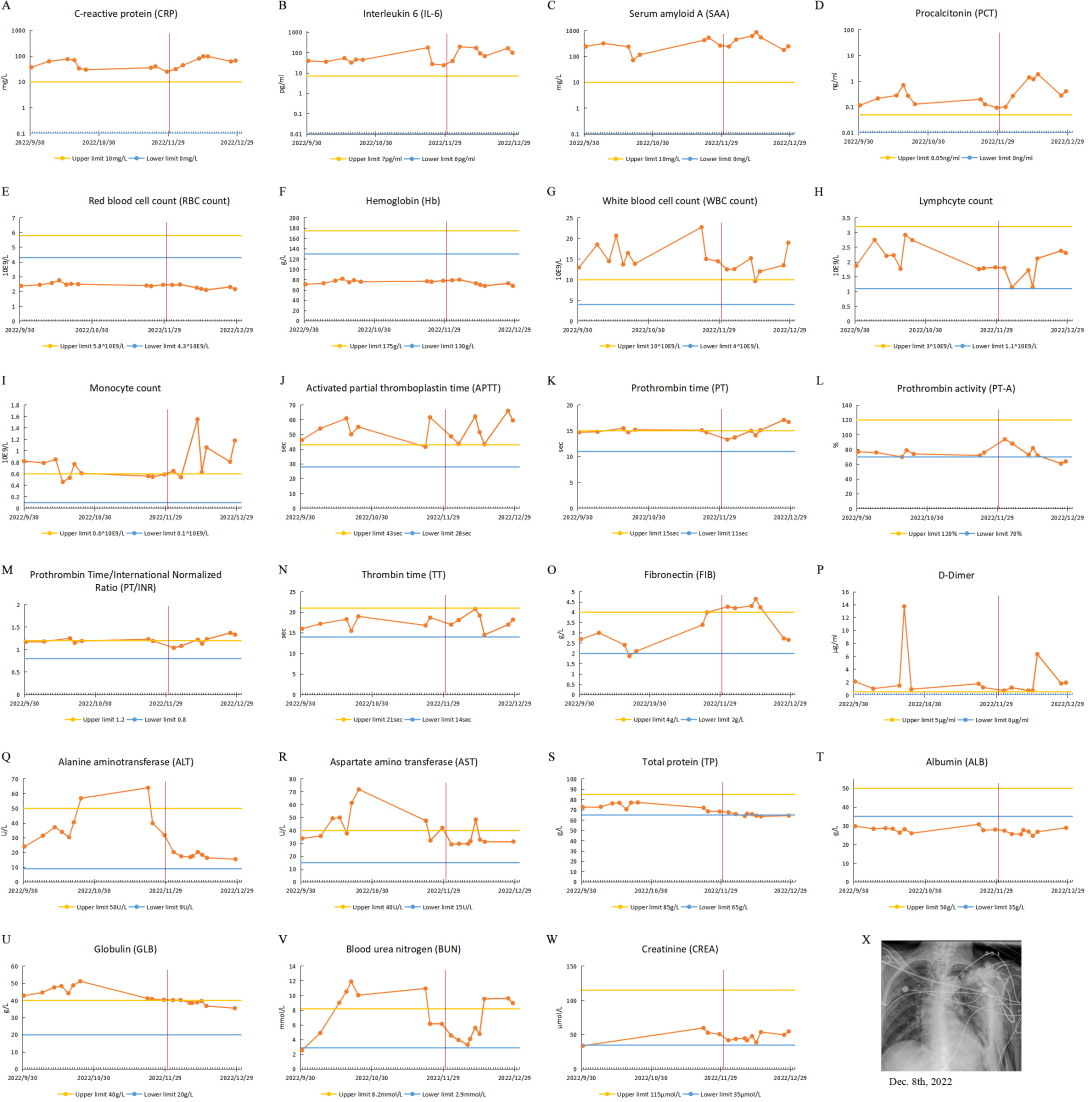
Clinical test results of case 2 from September 30, 2022, to December 31, 2022. **(A-W)** Test results of patient’s blood specimens. The horizontal axis represents the dates, and the solid orange dots indicate the test results at the corresponding time points. The red vertical line in the figure corresponds to November 30, 2022, when the city of Guangzhou ended closed-off management. **(A-D)** Infection-related test results: CRP, IL-6, PCT and SAA. **(E-I)** Routine blood test results: RBC, Hb, WBC, monocyte count, lymphocyte count. **(J-P)** Coagulation-related test results: APTT, PT, PT-A, PT/INR, TT, FIB and D-Dimer. **(Q, R)** Liver function-related test results: ALT and AST. **(S-U)** Serum protein-related test results: TP, ALB and GLB. **(V, W)** Renal function-related test results: BUN and CREA. **(X)** CXR test result.

He was diagnosed with bacterial pneumonia with carbapenem-resistant Klebsiella pneumoniae (CRKP) and Mycobacterium pneumoniae infections. His physical examination showed hyperresonance on percussion in bilateral lungs, increased breath sounds, audible moist rales, and no dry rales. Some of the clinical laboratory findings are shown in **Figure 2**. The examination results suggested leukocytosis, neutrophilia and elevated infection markers, and sputum culture and alveolar lavage fluid genetic testing showed CRKP (sensitive to ceftazidime avibactam, mediated to colistin), Mycobacterium tuberculosis complex group 747 sequences, and Mycobacterium abscessus 1035 sequences. He was treated with ventilator-assisted ventilation, sputum reduction, bronchoscopic aspiration and was also given meropenem, amikacin, tigecycline and mucomycin nebulization to fight infection. During treatment, there was impairment of liver function, which might be a side effect of tigecycline drug therapy. He was discharged on 26 October 2022.

He was readmitted to the hospital on 21 November 2022, due to cough, sputum, and decreased blood oxygen saturation. His diagnosis was bacterial pneumonia with CRKP, CRAB and Mycobacterium tuberculosis infection. Some of laboratory findings are also shown in the **Figure 2**. Sputum culture and genetic testing of alveolar lavage showed CRKP, CRAB (mediated to colistin), and Mycobacterium pneumoniae. CXR showed thickened, increased, and blurred texture in both lungs, localized grid-like changes, with multiple scattered speckled and flocculent slightly hyperdense blurred shadows in bilateral lungs, predominantly in the left lung. The ORF1ab Gene and N Gene assays for the 2019-nCoV-RNA were performed using RT-qPCR, and he was tested negative for the SARS-CoV-2 during hospitalization. He was treated with cefoperazone sulbactam, amikacin, meropenem and fluconazole and was discharged on 31 December 2022.

During hospitalization, the patient was also not infected with SARS-CoV-2. Interestingly, during hospitalization, the patient’s globulin level was above the upper reference limit before 30 November 2022, and then the globulin level gradually decreased but was still above the lower limit of the reference value. A follow-up phone call after discharge from the hospital revealed that he remained uninfected with SARS-CoV-2.

## 3 Discussion

We report two hospitalized cases of bacterial pneumonia with high globulin levels who were not infected with SARS-CoV-2 in Guangzhou, China. At the end of the closed-off management in Guangzhou city on November 30, 2022, their globulin levels were above the upper limit of the reference value. After ending of closed-off management, *i.e.*, exposure to SARS-CoV-2, their globulin levels all gradually decreased but remained above the lower reference limit.

When the lungs are invaded by bacteria causing pneumonia, the body’s immune response, particularly the mucosal immune response, is triggered to fight off the infection (13, 14). The innate immune response acts as the initial defense, without requiring prior recognition of specific pathogens, while the mucosal barrier prevents pathogen entry. In response to invasion, the immune system triggers inflammation, releasing cytokines and chemokines to recruit other immune cells. Macrophages, neutrophils, and other cells are mobilized to engulf and destroy bacteria through phagocytosis, aided by enzyme release and reactive oxygen species. The adaptive immune system, involving T cells and B cells, also contributes. T cells help regulate the immune response and activate immune cells, while B cells produce antibodies that bind to bacteria, marking them for destruction through opsonized phagocytosis or complement-mediated lysis, thus limiting pathogen spread.

The body’s immune system mounts a complex and fine-tuned response to the infection of SARS-CoV-2 (15–17). Upon infection with SARS-CoV-2 via the respiratory tract, the body initiates a local immune response. Immune cells such as macrophages and monocytes are recruited to respond to the infection by releasing cytokines and generating an adaptive T-cell and B-cell immune response. CD8+ T-cells are able to directly attack and kill virus-infected cells, whereas CD4+ T-cells are essential for turning on the function of CD8+ T-cells and B-cells. In addition, CD4+ T-cells are responsible for the production of cytokines that drive the recruitment of immune cells. Activated B-cells produce specific antibodies to neutralize SARS-CoV-2.

Antibodies against other viruses, such as human immunodeficiency virus (HIV), middle east respiratory syndrome coronavirus (MERS-CoV), and dengue virus, can also neutralize SARS-CoV-2 due to cross-reactivity (18–21). Interestingly, BCG vaccination (attenuated strain of tubercle bacillus Mycobacterium) improves the patient’s immunity against SARS-CoV-2 infection and improves prognosis (22). Several peptides of BCG proteins are highly homologous to that of the SARS-CoV-2 spike protein (22). BCG vaccination increases IgG antibodies in mice, which could neutralize the SARS-CoV-2 leading to the blocking of the SARS-CoV-2 infection (22). In this study, we also predicted antigenic epitopes in the RBD region of the SARS-CoV-2 spike protein, which is closely associated with viral invasion (22), using online software, and compared the predicted antigenic epitopes (“NGVGYQ” and “KIADYNYKLP”) (**Supplementary Figures 1**, **2**) with the protein sequences of Escherichia coli and Klebsiella pneumoniae. Many peptides of protein sequences of *Escherichia coli* and *Klebsiella pneumoniae* were found to be highly homologous to the predicted antigenic epitopes of the RBD region of the SARS-CoV-2 spike protein (**Supplementary Tables 1**-**3**). Further ELISA results showed that the monoclonal antibody against SARS-CoV-2 could bind the antigens of *Escherichia coli* and *Klebsiella pneumoniae* (**Supplementary Figure 3**). Therefore, the above reports and results suggest that antibodies produced by the body induced by bacterial infections or bacterial antigens, may neutralize SARS-CoV-2 viruses and hence blocks its invasion.

Elevated levels of Infection-associated tests include SAA, PCT, CRP, and IL-6 indicate the presence of infection (23). Two patients we studied had consistently high levels of infection-related tests, suggesting the presence of a persistent infection. Persistent existence of microorganisms on mucosal surfaces can lead to robust adaptive immunity including producing antibodies (24, 25). Hence, we hypothesized that the high globulin levels in the patients by the end of November 2022 were due to the body’s production of immunoglobulins induced by the persistent presence of infection in the body. And after exposure to SARS-CoV-2, the induced immunoglobulins, *i.e.* antibodies, cross-reacted with SARS-CoV-2, blocking the invasion and infection of SARS-CoV-2. A large amount of immunoglobulins in the body of patients are consumed to neutralize SARS-CoV-2, leading to a decrease in immunoglobulins and ultimately a corresponding decrease in the patient’s globulins.

This study also has limitations. With only two cases reported, the sample size is too small to draw conclusions that are generally applicable, and therefore not representative of the entire population of patients with bacterial pneumonia. Subsequent analyses are warranted to involve a larger number of cases, which will be retrospectively analyzed in collaboration with several healthcare centers to investigate the relationship between bacterial pneumonia, globulin levels, and SARS-CoV-2 infection, considering various factors such as age, gender, and others.

## 4 Conclusion

Bacterial pneumonia patients with high globulin levels were less susceptible to SARS-CoV-2. The immunoglobulins induced by bacterial infection could neutralize SARS-CoV-2 blocking the virus’s invasion and preventing infection. The results of the study are expected to provide new ideas for the prevention and treatment of SARS-CoV-2 infection. For example, when IVIG therapy is given to COVID-19 patients (26), the effect of using immunoglobulin extracted from patients with bacterial pneumonia may be more effective in treating SARS-CoV-2 infection compared to immunoglobulin sourced from healthy individuals.

## Data Availability

The raw data supporting the conclusions of this article will be made available by the authors, without undue reservation.
