# Supplementary material for: Bacterial pneumonia patients with elevated globulin levels did not get infected with SARS-CoV-2: two case reports
Source: Front Immunol. 2024 Aug 29;15:1404542. doi: 10.3389/fimmu.2024.1404542 (PMC11390513; doi:10.3389/fimmu.2024.1404542)

Supplementary Material

# Materials and Methods

**1.1 Reagents**

SARS-COV-2 spike protein Receptor Binding Domain (RBD) Human Monoclonal Antibody (Clone B7) (Cat# AF0507) and horseradish peroxidase labeled goat anti-human IgG (H+L) (Cat# A0201) were purchased from Beyotime (Shanghai, China). *Escherichia coli* (*E. coli*) (ATCC25922) and Klebsiella pneumoniae (ATCC700603) were laboratory preserved strains.

**1.2 *In silico* analysis**

The peptides of the antigenic epitope of the SARS-COV-2 spike protein RBD (P0DTC2|319-541, Uniprot) were predicted using the online software BepiPred (https://services.healthtech.dtu.dk/services/BepiPred-2.0/) Segments. Homologous peptide sequences were screened using the NCBI online Basic Local Alignment Search Tool (BLAST) (https://blast.ncbi.nlm.nih.gov/Blast.cgi), in which the database was selected as “Non-redundant protein sequences (nr) Non-redundant protein sequences (nr)”. When searching for homologous peptides of *E. coli*, the organism was selected as “*Escherichia coli* (taxid:562)” and 100 was set as the maximum number of aligned sequences to display. When searching for homologous peptides of Klebsiella pneumoniae, the organism was selected as “*Klebsiella pneumoniae* (taxid:573)”, and also set 100 as the maximum number of aligned sequences to display.

**1.3 Bacterial antigen preparation**

The strains of *E. coli* and *Klebsiella pneumoniae* stored in the ultra-low temperature refrigerator were inoculated on nutrient broth agar solid medium and placed at 37℃ for overnight culture. Single colonies were picked and placed in Nutrient Broth Liquid Medium and incubated overnight at 37℃ with shaking. Then the bacterial bodies were collected by centrifugation and resuspended by adding bacterial lysate (Cat#P0013, Beyotime, Shanghai, China) and protease inhibitor. After ultrasonic fragmentation of the bacteria, the supernatant was collected after centrifugation, which was the bacterial antigen. The concentration of bacterial antigen was detected by Bicinchoninic Acid Assay (BCA) (Beyotime, Shanghai, China).

**1.4 Enzyme linked immunosorbent assay （ELISA）**

1. Buffers and Reagents
2. Coating Solution: Dilute bacterial antigen to 10 µg/ml in PBS.
3. Blocking Buffer (10% FBS-PBS): Mix 10 mL FBS with 100 mL PBS, store at 2~8℃.
4. Sample Dilution Solution (10% FBS-PBST): Mix FBS with PBS and Tween 20 to 0.05% (v/v).
5. Wash Solution: Dilute 20X PBS with ultrapure water (1:20), add Tween 20 to 0.05% (v/v), store at room temperature.
6. Stopping Solution: Add 11 mL sulfuric acid to 90 mL ultrapure water, mix well, store at room temperature.
7. Experimental Procedure
8. Coating: Add 100 µL of coating solution per well in a 96-well plate, seal, refrigerate overnight (16~20h).
9. Washing: Discard liquid, wash with 300 µL wash solution 3 times, pat dry.
10. Blocking: Add 300 µL blocking buffer per well, seal, incubate at 37±1℃ for 1.5h±10min.
11. Washing: Discard liquid, wash with 300 µL wash buffer 3 times, pat dry.
12. 1st Antibody Incubation: Add 100 µL diluted SARS-COV-2 spike protein (RBD) Human Monoclonal Antibody (10µg/mL) per well, incubate at 37±1°C for 1h±10min.
13. Washing: Discard liquid, wash with 300 µL wash solution 3 times, pat dry.
14. 2nd Antibody Incubation: Add 100 µL diluted HRP-labeled goat anti-human IgG (H+L) (2.5ng/mL) per well, seal, incubate at 37±1°C for 1h±10min.
15. Washing: Discard liquid, wash with 300 µL wash solution 5 times, pat dry.
16. Color Development: Add 100 µL TMB solution per well, incubate at 37±1°C for 5~15 min.
17. Termination: Add 100 µL termination solution per well.
18. Reading: Read at 450nm using an enzyme meter.

# Results

**2.1 Prediction of antigenic epitopes of the RBD region of SARS-COV-2 spike Protein RBD**


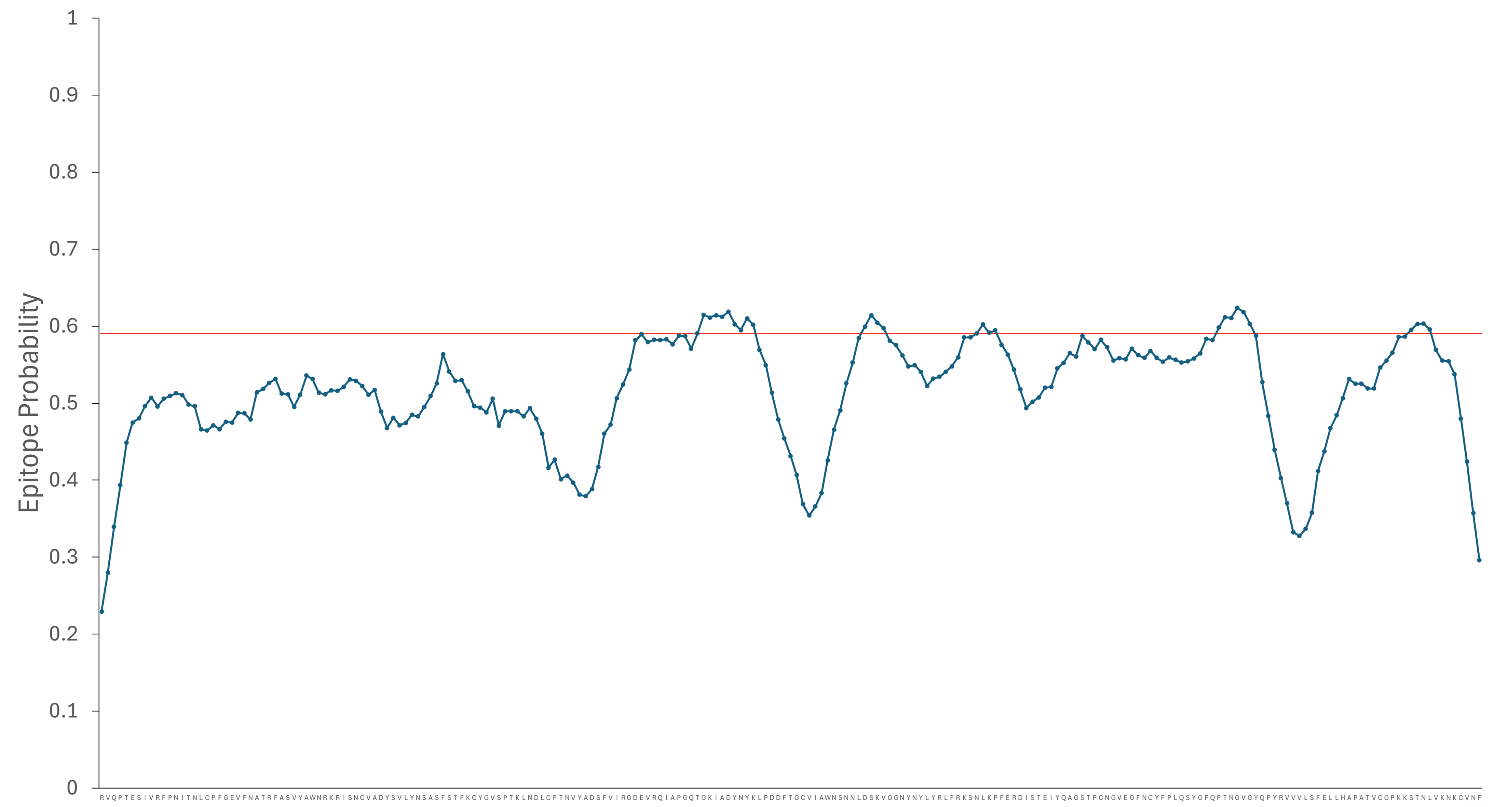
The epitope probability of individual amino acids of SARS-COV-2 spike protein RBD was predicted by BepiPred online software (**Supplementary Figure 1**). The threshold value was further set to 0.59, and multiple peptides were obtained (**Supplementary Figure 2**). Among them, there were two peptides with amino acid number longer than 5 amino acids, and the sequences were “NGVGYQ” and “KIADYNYKLP”, respectively.

**Supplementary Figure 1.** **Epitope probability of amino acids in the RBD region of the SARS-COV-2 spike protein.** The epitope probability of each amino acid was predicted using the online software BepiPred. The horizontal axis of the graph is the abbreviation of amino acids and the vertical axis is the corresponding epitope probability score for each amino acid.


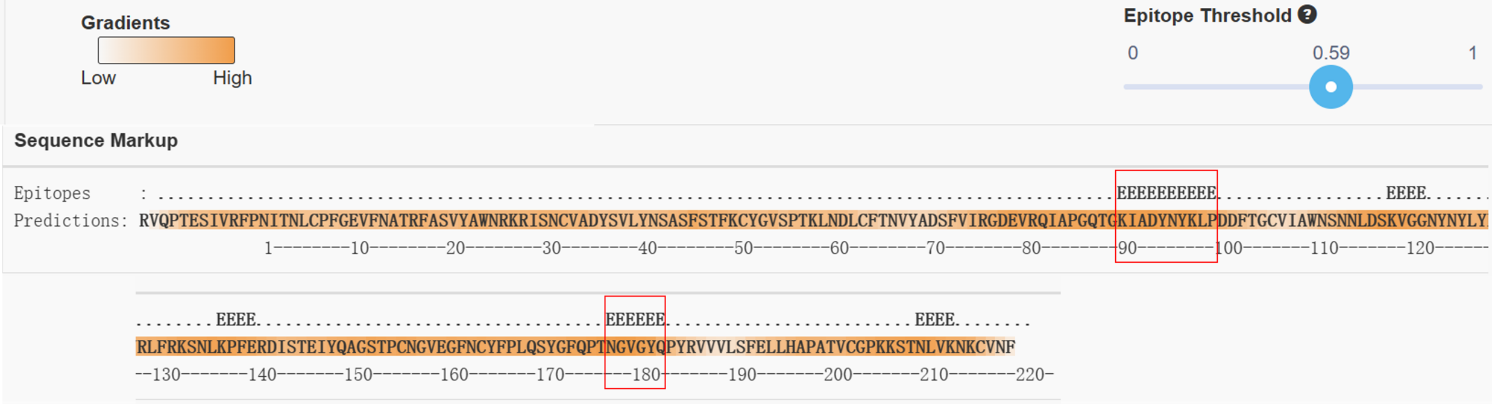


**Supplementary Figure 2.** **Screening for antigenic epitopes in the RBD region of the SARS-COV-2 spike protein.** The threshold was set to 0.59. “E”, epitope.

**2.2 Homologous sequence comparison of antigenic epitopes**

The above two peptides were used as query sequences for BLASTp (BLAST protein) analysis. The results showed that two proteins of *Escherichia coli* origin contained “NGVGYQ” sequences (**Supplementary Table 1**); 96 proteins of *E. coli* shared 6 or more amino acid sequences with “KIADYNYKLP” peptides (**Supplementary Table 2**); and 60 proteins of *Klebsiella pneumoniae* shared 6 or more amino acid sequences with “KIADYNYKLP” peptides (**Supplementary Table 3**). The above results indicate that bacterial antigens may contain antigenic epitopes in the RBD region of the SARS-COV-2 spike protein.

**Supplementary Table 1. Protein sequences of *E. coli* homologous to the antigenic epitope peptide “NGVGYQ”.** The underlined amino acids in the subject sequence are the amino acids that are homologous to “NGVGYQ”.

| Sequence ID | Name | Length | Subject sequence | Range | |
| --- | --- | --- | --- | --- | --- |
|  |  |  |  | From | To |
| HBA8094903.1 | hypothetical protein | 313 | NGVGYQ | 147 | 152 |
| STL35344.1 | putative lipoprotein | 127 | NGVGYQ | 60 | 5 |

**Supplementary Table 2. Protein sequences of *E. coli* homologous to the antigenic epitope peptide “KIADYNYKLP”.** The underlined amino acids in the subject sequence are the amino acids that are homologous to “KIADYNYKLP”.

| Sequence ID | Name | Length | Subject sequence | Range | |
| --- | --- | --- | --- | --- | --- |
|  |  |  |  | From | To |
| HBK9361186.1 | tyrosine-type recombinase/integrase | 418 | KIADFNYKKK | 223 | 232 |
| WP_072837441.1 | tyrosine-type recombinase/integrase | 418 | KIADFNYKKK | 223 | 232 |
| WP_136502523.1 | tyrosine-type recombinase/integrase | 418 | KIADFNYKKK | 223 | 232 |
| WP_059327688.1 | tyrosine-type recombinase/integrase | 418 | KIADFNYKKK | 223 | 232 |
| WP_134710081.1 | tyrosine-type recombinase/integrase | 418 | KIADFNYKKK | 223 | 232 |
| WP_247142187.1 | tyrosine-type recombinase/integrase | 418 | KIADFNYKKK | 223 | 232 |
| HBK9168668.1 | tyrosine-type recombinase/integrase | 418 | KIADFNYKKK | 223 | 232 |
| MDF8442345.1 | tyrosine-type recombinase/integrase | 403 | KIADFNYKKK | 223 | 232 |
| EKG7307435.1 | tyrosine-type recombinase/integrase | 396 | KIADFNYKKK | 201 | 210 |
| HAM0871651.1 | phage integrase family protein | 327 | KIADFNYKKK | 132 | 141 |
| HBM2004245.1 | tyrosine-type recombinase/integrase | 293 | KIADFNYKKK | 98 | 107 |
| HCP7916266.1 | tyrosine-type recombinase/integrase | 292 | KIADFNYKKK | 97 | 106 |
| EIE8788480.1 | tyrosine-type recombinase/integrase | 286 | KIADFNYKKK | 91 | 100 |
| EHE9928634.1 | phage integrase family protein | 284 | KIADFNYKKK | 89 | 98 |
| WP_203397250.1 | tyrosine-type recombinase/integrase | 281 | KIADFNYKKK | 223 | 232 |
| HDD1014963.1 | tyrosine-type recombinase/integrase | 280 | KIADFNYKKK | 85 | 94 |
| EFB2602298.1 | phage integrase family protein | 278 | KIADFNYKKK | 83 | 90 |
| EHB7762808.1 | phage integrase family protein | 236 | KIADFNYKKK | 88 | 97 |
| HAW9373473.1 | phage integrase family protein | 233 | KIADFNYKKK | 85 | 94 |
| EFN7081185.1 | site-specific integrase | 232 | KIADFNYKKK | 89 | 98 |
| ELE0713948.1 | tyrosine-type recombinase/integrase | 231 | KIADFNYKKK | 82 | 91 |
| HCN9942460.1 | tyrosine-type recombinase/integrase | 224 | KIADFNYKKK | 85 | 94 |
| MBN6258473.1 | site-specific integrase | 158 | KIADFNYKKK | 28 | 37 |
| WP_251302562.1 | tyrosine-type recombinase/integrase, partial | 140 | KIADFNYKKK | 10 | 19 |
| WP_249535265.1 | tyrosine-type recombinase/integrase, partial | 99 | KIADFNYKKK | 75 | 84 |
| WP_096957658.1 | hypothetical protein | 85 | QIADSNYKLP | 24 | 33 |
| ELH7516253.1 | hypothetical protein | 85 | QIADSNYKLP | 24 | 33 |
| ELQ2287565.1 | exodeoxyribonuclease V subunit beta | 1180 | RIADYNYDRH | 1125 | 1134 |
| ELP8181427.1 | exodeoxyribonuclease V subunit beta | 1180 | RIADYNYDRH | 1125 | 1134 |
| WP_255072979.1 | exodeoxyribonuclease V subunit beta | 1180 | RIADYNYDRH | 1125 | 1134 |
| EIK8037785.1 | exodeoxyribonuclease V subunit beta | 1180 | RIADYNYDRH | 1125 | 1134 |
| WP_255082854.1 | exodeoxyribonuclease V subunit beta | 1180 | RIADYNYDRH | 1125 | 1134 |
| EHR5184885.1 | exodeoxyribonuclease V subunit beta | 1180 | RIADYDYNLH | 1125 | 1134 |
| EJN7522062.1 | exodeoxyribonuclease V subunit beta | 1180 | RIADYNYDRH | 1125 | 1134 |
| WP_332977548.1 | exodeoxyribonuclease V subunit beta | 1180 | RIADYNYDRH | 1125 | 1134 |
| WP_097767798.1 | exodeoxyribonuclease V subunit beta | 1180 | RIADYNYDRH | 1125 | 1134 |
| HCP8732839.1 | exodeoxyribonuclease V subunit beta | 1180 | RIADYNYDRH | 1125 | 1134 |
| WP_337082332.1 | exodeoxyribonuclease V subunit beta | 1180 | RIADYNYDRH | 1125 | 1134 |
| HCO5008912.1 | exodeoxyribonuclease V subunit beta | 1180 | RIADYNYDRH | 1125 | 1134 |
| HBB0960486.1 | exodeoxyribonuclease V subunit beta | 1180 | RIADYNYDRH | 1125 | 1134 |
| WP_032261038.1 | exodeoxyribonuclease V subunit beta | 1180 | RIADYNYDRH | 1125 | 1134 |
| HBD5270552.1 | exodeoxyribonuclease V subunit beta | 839 | RIADYNYDRH | 784 | 793 |
| WP_149493573.1 | exodeoxyribonuclease V subunit beta, partial | 632 | RIADYNYDRH | 577 | 586 |
| HAL5927424.1 | beta-lactam sensor/signal transducer BlaR1 | 585 | SITDYNYKKP | 333 | 342 |
| MCH0695579.1 | type I-E CRISPR-associated protein Cas7/Cse4/CasC | 351 | KIADYLGKAKNYK | 93 | 105 |
| WP_240765908.1 | lipopolysaccharide 3-alpha-galactosyltransferase | 338 | SVIDYNYKLE | 14 | 23 |
| MQS28112.1 | class D beta-lactamase | 283 | SITDYNYKKP | 170 | 179 |
| HDI5968989.1 | PD-(D/E)XK nuclease family protein | 180 | RIADYNYDRH | 125 | 134 |
| WP_275940088.1 | M56 family metallopeptidase, partial | 131 | SITDYNYKKP | 114 | 123 |
| MEA0243251.1 | hypothetical protein | 688 | FNADYNYKIV | 496 | 505 |
| WP_289250969.1 | hypothetical protein | 688 | FNADYNYKIV | 496 | 505 |
| WP_149721578.1 | type VI secretion system contractile sheath large subunit, partial | 238 | KIAETNYKLG | 123 | 132 |
| MCU8652386.1 | tripartite tricarboxylate transporter substrate-binding protein | 140 | GIVDYNYKDF | 36 | 45 |
| EHI0969561.1 | beta-hydroxyacyl-ACP dehydratase | 182 | KIADYKYGEN | 26 | 35 |
| ELM0328793.1 | beta-hydroxyacyl-ACP dehydratase | 182 | KIADYKYGEN | 26 | 35 |
| WP_103767808.1 | 3-hydroxyacyl-ACP dehydratase FabZ family protein | 182 | KIADYKYGEN | 26 | 35 |
| HDX2959820.1 | beta-hydroxyacyl-ACP dehydratase | 182 | KIADYKYGEN | 26 | 35 |
| WP_096265855.1 | 3-hydroxyacyl-ACP dehydratase FabZ family protein | 182 | KIADYKYGEN | 26 | 35 |
| WP_096261755.1 | 3-hydroxyacyl-ACP dehydratase FabZ family protein | 182 | KIADYKYGEN | 26 | 35 |
| WP_000049968.1 | 3-hydroxyacyl-ACP dehydratase FabZ family protein | 182 | KIADYKYGEN | 26 | 35 |
| WP_089637822.1 | 3-hydroxyacyl-ACP dehydratase FabZ family protein | 182 | KIADYKYGEN | 26 | 35 |
| HAV0049104.1 | beta-hydroxyacyl-ACP dehydratase | 182 | KIADYKYGEN | 26 | 35 |
| MCS1314965.1 | beta-hydroxyacyl-ACP dehydratase | 182 | KIADYKYGEN | 26 | 35 |
| EHE2558689.1 | beta-hydroxyacyl-ACP dehydratase | 182 | KIADYKYGEN | 26 | 35 |
| HDW3967414.1 | beta-hydroxyacyl-ACP dehydratase | 182 | KIADYKYGEN | 26 | 35 |
| HBA9447509.1 | beta-hydroxyacyl-ACP dehydratase | 182 | KIADYKYGEN | 26 | 35 |
| EHV3971063.1 | beta-hydroxyacyl-ACP dehydratase | 182 | KIADYKYGEN | 26 | 35 |
| WP_089621163.1 | 3-hydroxyacyl-ACP dehydratase FabZ family protein | 182 | KIADYKYGEN | 26 | 35 |
| MCS0794180.1 | beta-hydroxyacyl-ACP dehydratase | 182 | KIADYKYGEN | 26 | 35 |
| HBI9907006.1 | beta-hydroxyacyl-ACP dehydratase | 182 | KIADYKYGEN | 26 | 35 |
| WP_235162168.1 | 3-hydroxyacyl-ACP dehydratase FabZ family protein | 182 | KIADYKYGEN | 26 | 35 |
| ELL8815513.1 | beta-hydroxyacyl-ACP dehydratase | 182 | KIADYKYGEN | 26 | 35 |
| WP_069906680.1 | 3-hydroxyacyl-ACP dehydratase FabZ family protein | 182 | KIADYKYGEN | 26 | 35 |
| EFK6853017.1 | beta-hydroxyacyl-ACP dehydratase | 182 | KIADYKYGEN | 26 | 35 |
| WP_274451179.1 | beta-hydroxyacyl-ACP dehydratase | 182 | KIADYKYGEN | 26 | 35 |
| WP_170897863.1 | 3-hydroxyacyl-ACP dehydratase FabZ family protein | 182 | KIADYKYGEN | 26 | 35 |
| HBB0036094.1 | beta-hydroxyacyl-ACP dehydratase | 182 | KIADYKYGEN | 26 | 35 |
| WP_044863223.1 | 3-hydroxyacyl-ACP dehydratase FabZ family protein | 182 | KIADYKYGEN | 26 | 35 |
| WP_257808835.1 | 3-hydroxyacyl-ACP dehydratase FabZ family protein | 182 | KIADYKYGEN | 26 | 35 |
| EFE2901144.1 | beta-hydroxyacyl-ACP dehydratase | 182 | KIADYKYGEN | 26 | 35 |
| EFQ3319469.1 | beta-hydroxyacyl-ACP dehydratase | 182 | KIADYKYGEN | 26 | 35 |
| HDS7478934.1 | beta-hydroxyacyl-ACP dehydratase | 182 | KIADYKYGEN | 26 | 35 |
| HDP9535913.1 | beta-hydroxyacyl-ACP dehydratase | 182 | KIADYKYGEN | 26 | 35 |
| EFG7696969.1 | beta-hydroxyacyl-ACP dehydratase | 182 | KIADYKYGEN | 26 | 35 |
| WP_000049964.1 | 3-hydroxyacyl-ACP dehydratase FabZ family protein | 182 | KIADYKYGEN | 26 | 35 |
| EGG0503365.1 | beta-hydroxyacyl-ACP dehydratase | 182 | KIADYKYGEN | 26 | 35 |
| WP_124848558.1 | 3-hydroxyacyl-ACP dehydratase FabZ family protein | 182 | KIADYKYGEN | 26 | 35 |
| ELQ4132432.1 | beta-hydroxyacyl-ACP dehydratase | 182 | KIADYKYGEN | 26 | 35 |
| EIT3802830.1 | beta-hydroxyacyl-ACP dehydratase | 182 | KIADYKYGEN | 26 | 35 |
| WP_227455397.1 | 3-hydroxyacyl-ACP dehydratase FabZ family protein | 182 | KIADYKYGEN | 26 | 35 |
| MED7528453.1 | 3-hydroxyacyl-ACP dehydratase FabZ family protein | 182 | KIADYKYGEN | 26 | 35 |
| WP_262824410.1 | 3-hydroxyacyl-ACP dehydratase FabZ family protein | 182 | KIADYKYGEN | 26 | 35 |
| HAH5461688.1 | beta-hydroxyacyl-ACP dehydratase | 182 | KIADYKYGEN | 26 | 35 |
| HEG1720469.1 | beta-hydroxyacyl-ACP dehydratase | 182 | KIADYKYGEN | 26 | 35 |
| EFC0989222.1 | beta-hydroxyacyl-ACP dehydratase | 182 | KIADYKYGEN | 26 | 35 |
| EFO1905504.1 | beta-hydroxyacyl-ACP dehydratase | 182 | KIADYKYGEN | 26 | 35 |

**Supplementary Table 3. Protein sequences of *Klebsiella pneumoniae* homologous to the antigenic epitope peptide “KIADYNYKLP”.** The underlined amino acids in the subject sequence are the amino acids that are homologous to “KIADYNYKLP”.

| Sequence ID | Name | Length | Subject sequence | Range | |
| --- | --- | --- | --- | --- | --- |
|  |  |  |  | From | To |
| HBX6151349.1 | site-specific integrase | 418 | KIADFNYKKK | 223 | 232 |
| WP_326380537.1 | EpsG family protein | 373 | KISDYNYLLT | 213 | 222 |
| WP_255969706.1 | alternative oxidase, partial | 66 | KIAIDYYKLP | 24 | 33 |
| MCY0683100.1 | transcription elongation factor GreB | 82 | ENADYNYKKK | 22 | 31 |
| HDS8679177.1 | tail fiber domain-containing protein | 1305 | TDADYNYNLF | 800 | 809 |
| WP_040177168.1 | MULTISPECIES: tail fiber domain-containing protein | 1253 | TDADYNYNLF | 748 | 757 |
| WP_080922301.1 | tail fiber domain-containing protein | 1253 | TDADYNYNLF | 748 | 757 |
| HEB8708284.1 | tail fiber domain-containing protein | 1253 | TDADYNYNLF | 748 | 757 |
| WP_117065202.1 | tail fiber domain-containing protein | 1151 | TDADYNYNLF | 645 | 654 |
| ELN9684907.1 | tail fiber domain-containing protein | 1092 | TDADYNYNLF | 587 | 596 |
| MBZ2019314.1 | hypothetical protein | 815 | TDADYNYNLF | 748 | 757 |
| WP_264958767.1 | hypothetical protein, partial | 291 | TDADYNYNLF | 154 | 163 |
| WP_334262400.1 | hypothetical protein, partial | 273 | TDADYNYNLF | 184 | 193 |
| WP_184808128.1 | hypothetical protein, partial | 233 | TDADYNYNLF | 141 | 150 |
| WP_221016222.1 | hypothetical protein, partial | 230 | TDADYNYNLF | 121 | 130 |
| ELJ9568829.1 | hypothetical protein | 182 | TDADYNYNLF | 115 | 124 |
| WP_180976841.1 | hypothetical protein, partial | 174 | TDADYNYNLF | 133 | 142 |
| MCM6206979.1 | hypothetical protein | 221 | KLADYKYGLP | 70 | 79 |
| SSN07285.1 | putative phage repressor protein CI | 239 | SDADYNGYKL | 122 | 131 |
| KAA5707046.1 | acetate CoA-transferase YdiF, partial | 186 | KIAAYNYPQG | 57 | 66 |
| KAA5800083.1 | hypothetical protein F3G47_31140, partial | 140 | KIAAYNYPQG | 110 | 119 |
| WP_117270177.1 | hypothetical protein | 650 | LYMDYNYKAP | 102 | 111 |
| HDE1929563.1 | hypothetical protein | 650 | LYMDYNYKAP | 102 | 111 |
| HBV4971031.1 | hypothetical protein | 650 | LYMDYNYKAP | 102 | 111 |
| HCD4106160.1 | integrating conjugative element protein | 471 | KIADYAYSSQ | 161 | 170 |
| WP_047670511.1 | hypothetical protein | 460 | FSADYHYDLP | 169 | 178 |
| HBR3116010.1 | DUF4297 domain-containing protein | 408 | KIGDYRSNYK | 69 | 78 |
| HDZ1491572.1 | DUF4297 domain-containing protein | 408 | KIGDYRSNYK | 69 | 78 |
| HEJ0136942.1 | DUF4238 domain-containing protein | 346 | KIEEYKKLNYKL | 220 | 231 |
| WP_061891527.1 | DUF4238 domain-containing protein | 346 | KIEEYKKLNYKL | 220 | 231 |
| HBR1373093.1 | DUF4238 domain-containing protein | 346 | KIEEYKKLNYKL | 220 | 231 |
| HCB0303940.1 | hypothetical protein | 339 | FSADYHYDLP | 48 | 57 |
| HCF7941383.1 | hypothetical protein | 326 | FSADYHYDLP | 169 | 178 |
| SCA95880.1 | putative rhamnosyltransferase | 308 | KIAGYNLGKRYKKLP | 279 | 293 |
| HBX6293504.1 | glycosyltransferase family 2 protein | 302 | KIAGYNLGKRYKKLP | 273 | 287 |
| HBT4306584.1 | hypothetical protein | 284 | KIEEYKKLNYKL | 158 | 169 |
| HDZ0817408.1 | hypothetical protein | 283 | KIEEYKKLNYKL | 157 | 168 |
| HBR3248080.1 | hypothetical protein | 282 | KIEEYKKLNYKL | 164 | 175 |
| WP_126494887.1 | MULTISPECIES: hypothetical protein | 271 | KIAKYNYSAV | 127 | 136 |
| WP_226347301.1 | ABC-three component system protein | 217 | KIGDYRSNYK | 69 | 78 |
| SSW79765.1 | Uncharacterised protein | 156 | SRADYNDIYKL | 55 | 65 |
| WP_135735319.1 | hypothetical protein | 121 | NKAGFNYKLP | 45 | 54 |
| WP_107326871.1 | hypothetical protein | 121 | NKAGFNYKLP | 45 | 54 |
| HBQ6862584.1 | hypothetical protein | 116 | NKAGFNYKLP | 45 | 54 |
| HBW4509642.1 | hypothetical protein | 104 | NKAGFNYKLP | 45 | 54 |
| WP_289490238.1 | hypothetical protein, partial | 76 | FIADYIYKPISHDLP | 41 | 55 |
| MDH8560358.1 | hypothetical protein | 74 | KIADYAYVLE | 28 | 37 |
| HBW7288909.1 | hypothetical protein | 864 | NIADYLENFK | 712 | 721 |
| WP_223325984.1 | ATP-binding protein | 864 | NIADYLENFK | 712 | 721 |
| WP_060598585.1 | ATP-binding protein | 864 | NIADYLENFK | 712 | 721 |
| WP_330764609.1 | ATP-binding protein | 864 | NIADYLENFK | 712 | 721 |
| WP_073901605.1 | ATP-binding protein | 864 | NIADYLENFK | 712 | 721 |
| WP_135717941.1 | ATP-binding protein | 864 | NIADYLENFK | 712 | 721 |
| HBY9060462.1 | ATP-binding protein | 864 | NIADYLENFK | 712 | 721 |
| EKX1847150.1 | ATP-binding protein | 859 | NIADYLENFK | 707 | 716 |
| HCC2962460.1 | sensor histidine kinase | 823 | NIADYLENFK | 712 | 721 |
| MCS6058469.1 | ATP-binding protein | 822 | NIADYLENFK | 712 | 721 |
| HBY9877541.1 | ATP-binding protein | 761 | NIADYLENFK | 609 | 618 |
| EKU8495177.1 | ATP-binding protein | 749 | NIADYLENFK | 597 | 606 |
| HCD5770023.1 | sensor histidine kinase | 544 | NIADYLENFK | 392 | 401 |

**2.3 Monoclonal antibodies against the RBD region of the SARS-COV-2 spike protein cross-react with bacterial antigens**

The above *in silico* analysis data suggested antigenic homology. We further verified the cross-reactivity of the antibodies by ELISA. In the control group, we used PBS instead of 1st antibody, and other conditions were the same as the experimental group. It was found that the 450nm OD values of the experimental group of *E. coli* and *Klebsiella pneumoniae* were larger than their corresponding control groups (**Supplementary Figure 3**). These results suggest that the monoclonal antibody against the RBD region of SARS-COV-2 spike protein binds the bacterial antigens of *E. coli* and *Klebsiella* pneumoniae.

**Supplementary Figure 3. ELISA results.** Monoclonal Antibody, the monoclonal antibody against the RBD region of SARS-COV-2 spike protein. *, *P*<0.05.
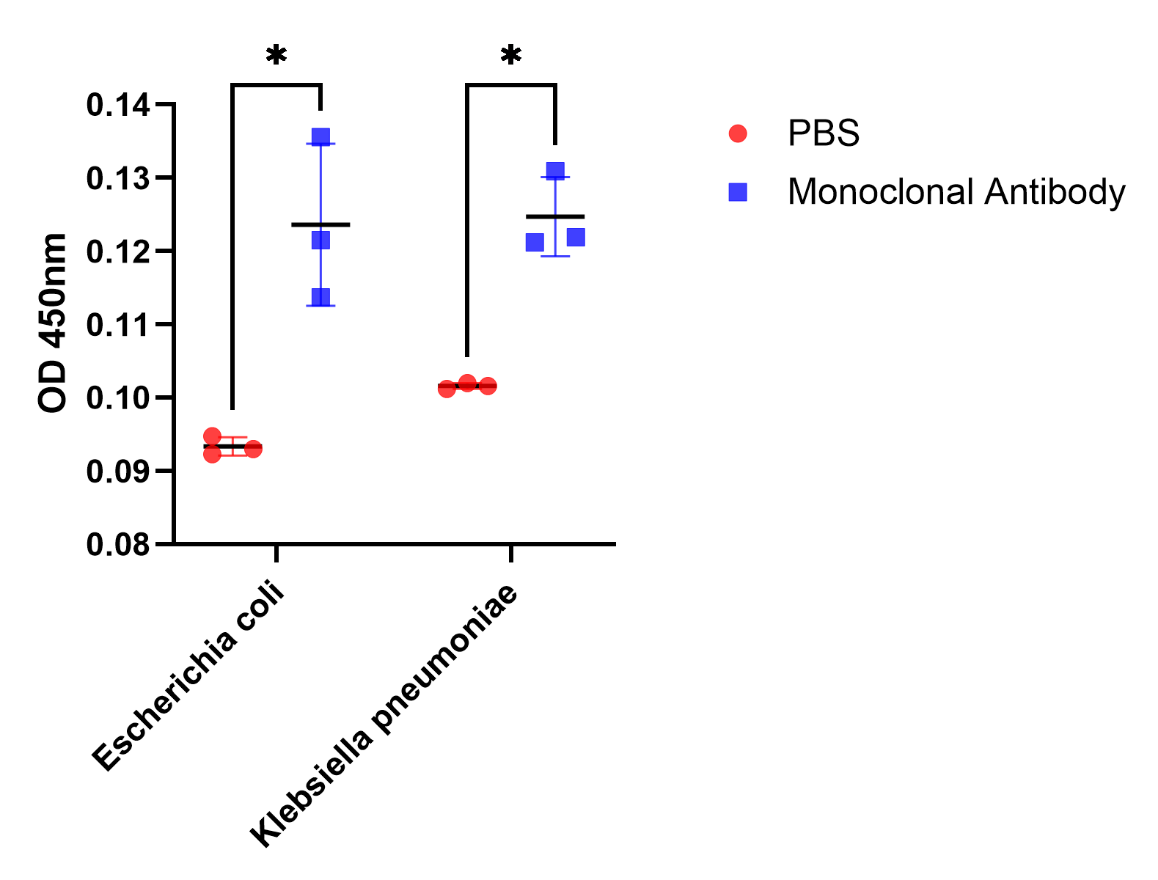

Supplement: Supplementary file 1 [file DataSheet1.docx]
